# Rapid Determination of SARS-CoV-2 antibodies using a bedside, point-of-Care, serological test

**DOI:** 10.1080/22221751.2020.1826892

**Published:** 2020-10-07

**Authors:** Laurent Dortet, Cecile Emeraud, Christelle Vauloup-Fellous, Mouna Khecharem, Jean-Baptiste Ronat, Nicolas Fortineau, Anne-Marie Roque-Afonso, Thierry Naas

**Affiliations:** a Service de Bactériologie-hygiène, Hôpital Bicêtre, Inserm U1184; LabEx LERMIT, Université Paris-Saclay, APHP Paris-Saclay, Le Kremlin-Bicêtre, France; b Service de Virologie, Hôpital Paul-Brousse, Inserm U1193; Université Paris-Saclay, APHP Paris-Saclay, Villejuif, France; c Médecins Sans Frontières, Mini-Lab project, Paris, France

**Keywords:** COVID-19, serology, diagnosis, rapid test, Diagnostics, bedside

## Abstract

**Background**: Several serological tests for SARS-CoV-2 have been developed or use, but most have only been validated on few samples, and none provide medical practitioners with an easy-to-use, self-contained, bedside test with high accuracy. **Material and methods**: Two-hundred fifty-six sera from 101 patients hospitalized with SARS-CoV-2 infection (positive RT–PCR) and 50 control sera were tested for IgM/IgG using the NG-Test IgM-IgG COVID all-in-one assay. The seroconversion dynamic was assessed by symptom onset and day of RT–PCR diagnosis. **Results:** Among the SARS-CoV-2 infected patients, positive IgG and/or IgM result was observed for 67.3% of patients (68/101), including 17 (16.8%) already positive at the day of RT–PCR, and 51 (50.5%) with observable seroconversion, and 32.7% (33/101) remained negative as subsequent sampling was not possible (patient discharge or death). The sensitivity increased with the delay between onset of symptoms and sampling, going from 29.1%, 78.2% and 86.5% for the time periods of 0-9-, 10-14- and >14-days after the onset of symptoms, respectively. Cumulative sensitivity, specificity, Positive Predictive Value and Negative Predictive Value were 97.0%, 100%, 100% and 96.2%, respectively 15-days after the onset of symptoms. No difference in seroconversion delay was observed regardless of whether patients received ventilation. **Conclusions:** The NG-test is a bedside serological assay that could serve as a complementary source of diagnostic information to RT–PCR and chest imaging. It may also be useful to monitor immunological status of medical and non-medical workers during the ongoing pandemic, and the general population after social distancing measures have eased.

## Introduction

Since first being reported by the Chinese Centre for Disease Control and Prevention (CCDC) on January 9th, SARS-CoV-2 has become a global pandemic, straining the world’s health systems with an exponentially increasing number of acute SARS-CoV-2 respiratory failures [1–4]. As of this writing, over 10.9 million COVID-19 cases have occurred, with over 521,669 deaths in more than 188 countries [5]. Clinical manifestations of SARS-CoV-2 infection are highly nonspecific, including respiratory symptoms, fever, cough, and dyspnoea, but patients can also develop pneumonia, acute respiratory failure, and other serious complications [6–8]. In the absence of preventive or curative treatments, social distancing measures are at the forefront of the unprecedented efforts to contain the disease. Moving forward, however, reliably detecting infections will become central to monitoring the pandemic, informing health policy, rapidly responding to events as they evolve, and mitigating disease transmission [9–11]. Moreover, better virologic information from infected individuals could help estimate the size of the viral reservoir, more complicated for SARS-CoV-2 because of pre-symptomatic and asymptomatic carriers who are nevertheless contagious and may be responsible for two-thirds of viral propagation [12]. Suppressing transmission from these cases will considerably reduce the total caseload and transmission of SARS-CoV-2 [13].

Diagnostics will thus need to rapidly scale to stop the evolving pandemic. Yet the current gold standard technique, real-time reverse transcription-polymerase chain reaction (rRT-PCR), (whose protocol has been available online since 17 January 2020) has substantial limitations. It requires specialized, expensive laboratory equipment, is often only located in laboratories with biosafety level ≥2, and may require sample transportation that can delay results for 2–3 days (in which time COVID-19 suspects may wait in dedicated “waiting” wards where they may further expose others patients and health workers) [10,14,15]. For SARS-CoV-2, RT–PCR testing also uses naso-pharyngeal swab samples that can be complicated to obtain, pose considerable risk to health care providers with insufficient personal protective equipment (PPE), and produce false-negative results in up to 30% of confirmed COVID-19 patients [16–18]. Chest radiography (CXR) and computed tomography (CT) scans show promise as ways to overcome PCR tests’ lack of sensitivity. However, in areas where flu or other respiratory viruses are still circulating, these chest imaging technologies may reveal images indicative of viral pneumonia [19]. CT and CXR equipment also demand sterilization and personal protective measures for staff after each use.

Serological confirmation of SARS-CoV-2 could thus provide an important complementary source of diagnostic information and help to estimate the proportion of individuals who have previously been infected in a population [10,17]. Serological response has a long signature (several months for IgM and IgG responses; longer for IgG titres), whereas molecular tests are positive only in actively infected individuals over a narrow period (PCR: 9.5 days to a few weeks after symptom onset) [20,21–24]. The time to seroconversion post-infection is also estimated to be only 7–14 days after symptoms appear [10,22,25]. Serological assays for COVID-19 are currently available but, in most cases, neither their analytical performance nor their usefulness in a clinical setting has been evaluated or has been evaluated on an extremely small number of sera [26]. Among the over 170 COVID-19 antibody detection tests listed on the FIND website [27] as being in some stage of development or use, none are a self-contained, point-of-care (PoC) testing device that is rapid, robust, cost-efficient, and could be used on-site or by the patients themselves. We retrospectively analyzed such a serological test in a cohort of French patients in Paris to assess its diagnostic accuracy and clinical utility for patient management.

## Materials and methods

### Patients and sera tested

From March 11–23^rd^, 256 sera were collected from 101 RT–PCR confirmed patients during COVID-19 specific consultations or while patients were in the emergency department. Among these patients, 82.2% (83/101) were hospitalized: 13.3% (11/83), were directly admitted to the ICU, 86.7% (72/83) were in COVID-19 wards, and 17.8% (18/101) were discharged. SARS-CoV-2 testing was performed on the same day as the patient’s consultation using rRT-PCR on respiratory tract samples [15]. The date of symptom onset, RNA testing results, and personal demographic information were obtained from clinical records.

A total of 50 samples were also collected to assess specificity: 24 sera collected from September-October 2017, before the COVID pandemic, 4 from patients with respiratory symptoms that were RT–PCR negative for SARS-CoV-2 but positive for common coronaviruses (Coronavirus HKU1 (n = 2), NL63 (n = 1), 229E (n = 1)) using Respiratory 2 FilmArray (Biofire, bioMérieux, France), and from 22 healthy volunteers without any respiratory symptoms. The latter were tested directly using a drop of whole blood.

### Molecular testing

Nasopharyngeal samples (eSwabs™-Virocult, Copan, Italy) were collected from all patients with COVID-19 symptoms. Real-time RT–PCR targeting RNA-dependent RNA polymerase and E genes were used to detect the presence of SARS-CoV-2 as described by Corman and colleagues [15].

### NG-Test IgM-IgG COVID All-in-one lateral flow immunoassay

The NG-Test IgM-IgG COVID All-in-One cassette (NG Biotech, Guipry France) is a qualitative, membrane-based immunoassay for the detection of IgG and IgM specific anti-SARS-CoV-2 antibodies using whole blood (from venipuncture or finger prick), serum, or plasma (Figure S1). The assay contains anti-human IgM and anti-human IgG as the capture reagent, and SARS-CoV-2 (Nucleocapsid protein) antigen gold particles as the detection reagent. A goat anti-mouse IgG is used in the control line system (Figure S1). The NG-Test IgM-IgG COVID All-in-One cassette was performed according to the manufacturer’s instructions by adding either ten µl of serum or a drop of blood (after finger puncture) into the sample port, followed by delivering a dilution buffer using the release button. Results were read after 15 min according to the manufacturer’s recommendations (Figure S1).

### Statistical analysis

Serological data from the immunoassay were compared to RT–PCR results. The sensitivity, specificity, positive predictive value, and negative predictive values were calculated with their respective confidence intervals (95% CI) using the free software vassarStats [28].

#### Ethics

The use of samples was reviewed and approved by the local Ethics Committee under CPP N° CO-15-000.

## Results

### Patient and sera Characteristics

Among 101 COVID-19 patients hospitalized from 11–23 March 2020, the median age was 58 years (IQR, 35-61) and the male/female ratio was 1.46. Among these individuals, 10.9% (11/101) were critically ill and required immediate hospitalization in the ICU and 17.8% (18/101) were discharged. The others were hospitalized in a dedicated COVID ward. Over the study period, a total of 36 patients (35.6%) were transferred to the ICU and ventilated (including 11 patients hospitalized in the ICU), of whom 25% (9/36) died an average 5.9 days (± 0.9) after ICU admission (range 3–10 days). On average, 2.6 sera were included per patient (Table S1).

**Figure 1.**
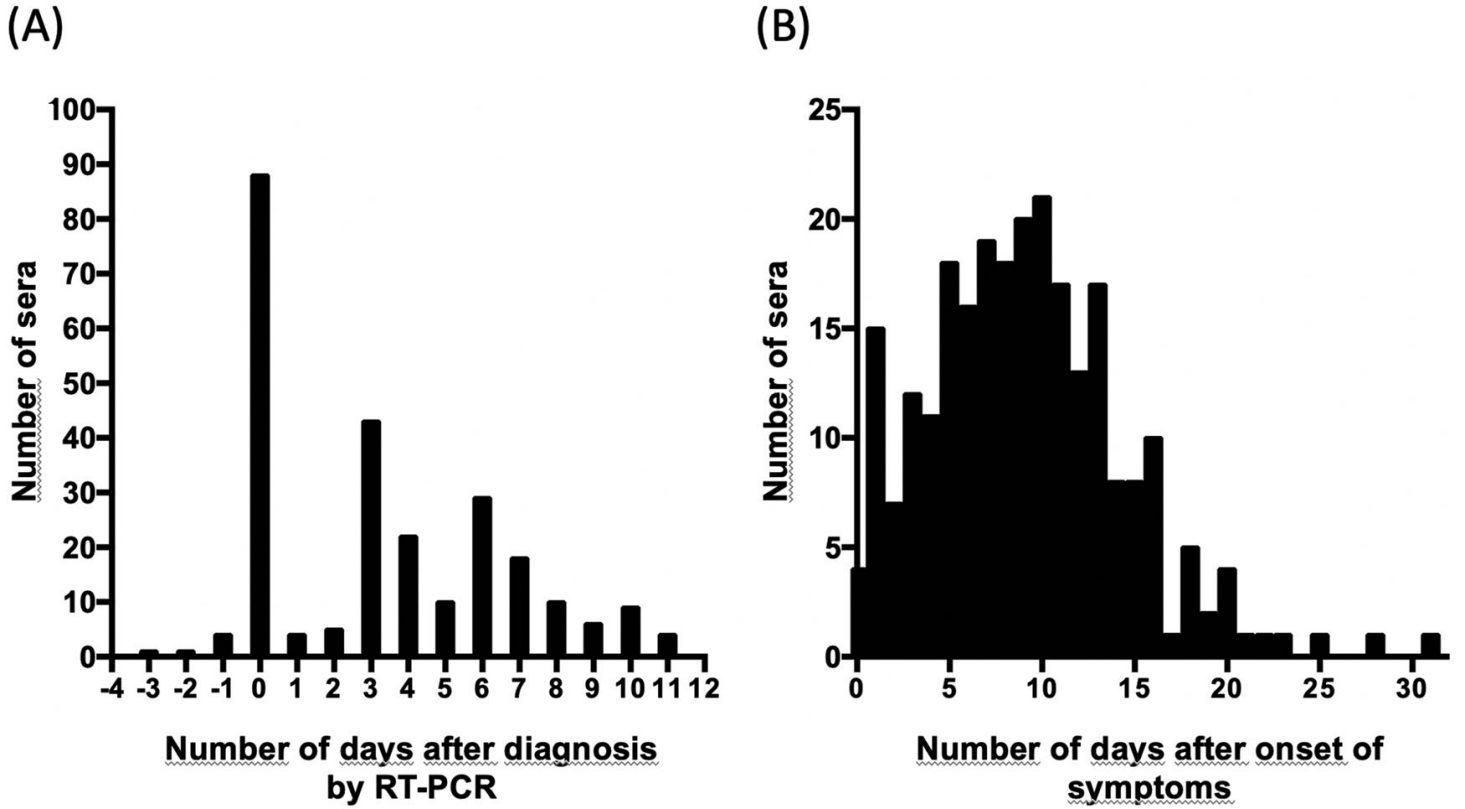
Distribution of sera included in this study. (A) Numbers of sera per day after diagnosis by RT-PCR; and (B) numbers of sera per day after onset of symptoms

For 97 patients, sera were available from the first day of hospitalization, when nasopharyngeal sampling was performed for RT–PCR testing, until the eleventh day of hospitalization (Figure 1A). Most sera were sampled between day 0–15 after the onset of symptoms (85.5%, 219/256) but later sera, up to day 31, were also available (Figure 1B).

### Test results in infected patients and controls

All 50 COVID-19 negative control sera were negative for both IgG and IgM using the NG-Test IgM-IgG COVID All-in-One assay. Specifically, no cross-reactivity was detected in the 4 subjects with recent common coronavirus infections in the past 3-months.

A total of 256 serum samples collected during the study period (n = 101 patients) and were retrospectively tested for IgM/IgG against SARS-CoV-2 using the NG-Test IgM-IgG COVID All-in-One device.

**Figure 2.**
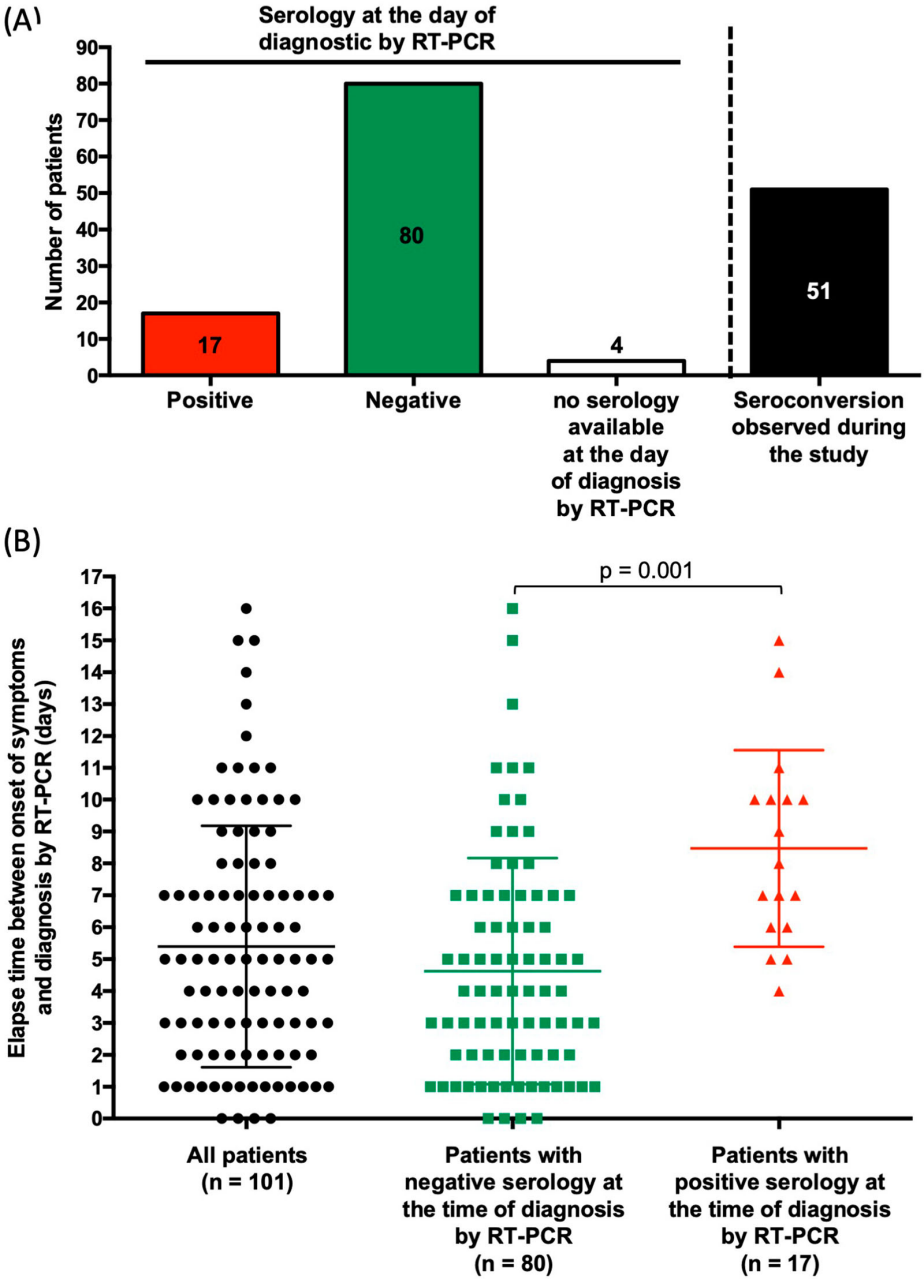
Characteristics of tested patients. (A) Serological status at the day of diagnosis by RT-PCR and seroconversion. (B) Elapse time between onset of symptoms and diagnostic by RT-PCR. Comparison was performed using Student t test with Welch correction. *p* < 0.05 was considered as significant.

Among SARS-CoV-2 infected patients, a positive result for IgG and/or IgM was observed for 67.3% of patients (68/101), including 51 (50.5%) with observable seroconversion on serial samples (Figure 2A and Table S2). For 17 patients (16.8%) IgM and/or IgG were already positive the day RT–PCR testing was performed, while 80 were negative and 4 had no serum available for testing (patients 1, 39, 74, and 98 in Table S2 and Figure 2A), though these 4 patients had sera that tested positive from 3 to 13 days after RT–PCR testing (Figure 2A and Table S2).

Among SARS-CoV-2 infected patients, 33 were negative for both IgG and IgM for the duration of the study period, as subsequent sampling was not possible. Eighteen patients were discharged from hospital with only one negative serology result available (only early sera from days 0–8 after becoming symptomatic), 2 patients died before the second sampling (at day 1 and 3 of symptoms), one patient died at day 8 with persistently negative serology (Table S2). Six patients were discharged with persistently negative serology before day 10. The last 6 patients were discharged at day 11, 14 and 18 with negative serology throughout (Table S2).

The average time between the onset of symptoms and receiving an RT–PCR result (essentially, admission at the hospital) was 5.4 (± 0.4) days (Figure 2B). Predictably, this delay was significantly higher in patients with positive serology when compared to those with negative serology at admission (4.6 ± 0.4 days *vs* 8.5 ± 0.7 days, *p* = 0.001) (Figure 2B).

### Seroconversion Dynamics

**Figure 3.**
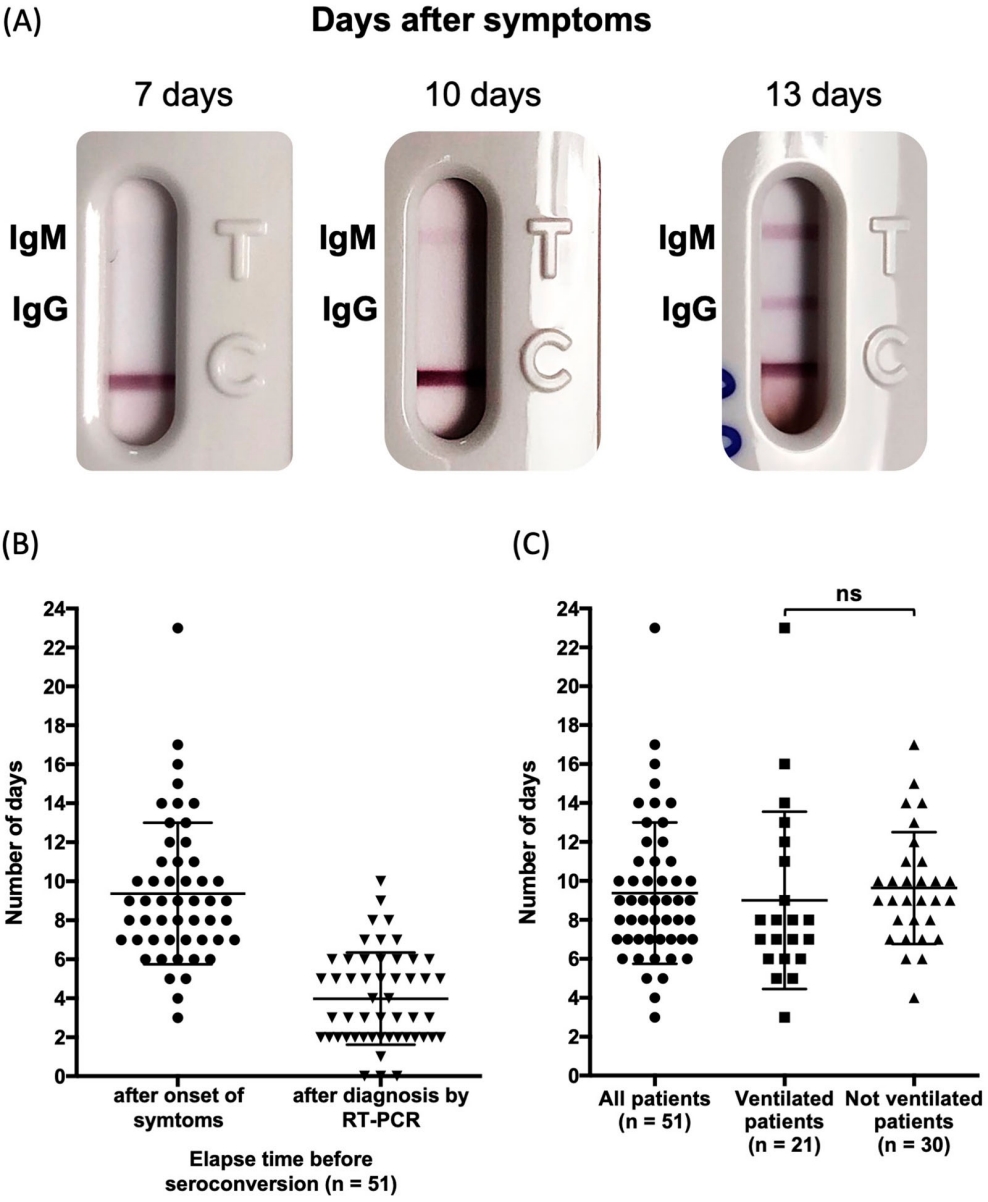
Seroconversion. (A) Representative results of a seroconversion with initial negative serum, appearance of IgM alone and IgM + IgG at days 7, 10 and 13, respectively; (B) Elapsed time for seroconversion after onset of symptoms and after diagnosis by RT-PCR; (C) Elapsed time for seroconversion in ventilated and none-ventilated patients. Statistically significance was determined using Student t test with Welch correction (*p* < 0.05 was considered as significant.). “ns” stands for not significant.

Seroconversion could be assessed for 51 patients with at least one negative serum followed by one or more positive sera (Figure 3A and Table S2). For these patients, the first sample was available early after the onset of symptoms: before day 5 in 25 patients, from day 5–8 in 13 patients, from day 9–10 in 4 patients, and from day 13–15 for 11 patients. Among these 51 patients with monitored seroconversion (with at least one negative serum followed by one or more positive sera), the change occurred 9.4 (± 0.5) days after the onset of the patient’s first symptoms, and 3.6 (± 0.4) days after RT–PCR testing (Figure 3B). No significant difference could be observed between ventilated (n = 21) and non-ventilated patients (n = 30) (9.6 ± 0.5 days vs 9.0 ± 1.0 days) (Figure 3C).

**Figure 4.**
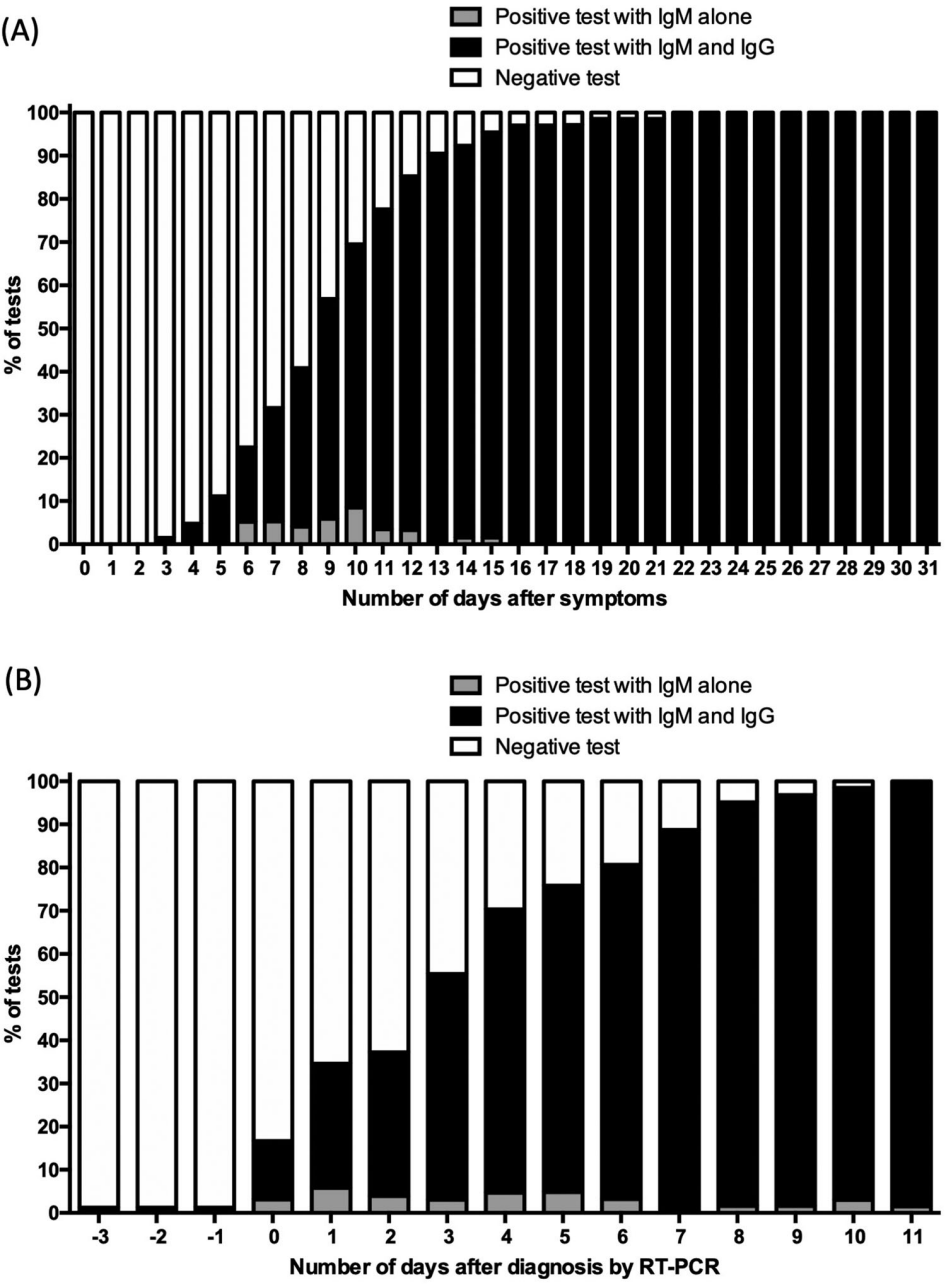
Cumulative incidence of seroconversion of IgG/M against SARS- CoV-2 among COVID-19 patients (A) after RT-PCR testing; and (B) after onset of first symptoms.

Positive IgM and IgG results in the first sample was observed for 17 patients, indicating seroconversion prior to hospital admission (Figure 2A). For most patients, both IgM and IgG appeared at the same time (Table S2). The typical sequential seroconversion with successive appearance of IgM and IgM + IgG could be observed for only 9 patients (Figures 3A and 4A). When IgM were observed alone, IgG appeared within one to two days (Table S2).

### NG-Test IgM-IgG COVID All-in-one performances

The overall sensitivity for IgM/IgG detection increased with the delay between onset of symptoms and sampling, being 29.1%, 78.2% and 86.5% for the time periods of day 0–9, day 10–14 and day >14 after the onset of symptoms, respectively (Table 1). These values were similar when the two immunoglobulins were considered individually.

**Table 1.** Performances of the NG-Test IgM-IgG COVID All-in-One assay according to time intervals from the onset of symptoms

Sensitivity according to the onset of symptoms [confidence interval at 95%]				Specificity
	D 0-9^a^	D 10–14	D>14	
IgM + IgG	29.1% (41/141) [21.9%–37.4%]	78.2% (61/78) [67.1%**–**86.4%]	86.5% (32/37) [70.4%**–**94.9%]	100% [91.7%**–**100%]
IgM	29.1% (41/141) [21.9%**–**37.4%]	78.2% (61/78) [67.1%**–**86.4%]	86.5% (32/37) [70.4%**–**94.9%]	100% [91.7%**–**100%]
IgG	24.8% (35/141) [18.1%**–**32.9%]	74.4% (58/78) [63.0%**–**72.1%]	86.5% (32/37) [70.4%**–**94.9%]	100% [91.7%**–**100%]

^a^ D: Day

The cumulative seroconversion curve with respect to the onset of symptoms showed that the rate for IgM/IgG reached >95% for 67 patients with sera available 15 days after symptom onset (Figure 4A, Table 2). The median time to IgM/IgG seroconversion was 8 days after symptom onset. For one patient, a pregnant woman, seroconversion occurred 22 days after she became symptomatic (Table 2 and S2).

**Table 2.** Cumulative sensitivity and specificity of the NG-Test IgM-IgG COVID All-in-One by day of symptom onset

Day after symptoms	N	Sensitivity		Specificity		PPV		NPV	
		%	CI95%	%	CI95%	%	CI95%	%	CI95%
**0**	80	**0**	0–5·3	**100**	91·1–100	**–**	**–**	**38·5**	30·2–47·4
**1**	77	**0**	0–5·9	**100**	91·1–100	**–**	**–**	**39·4**	30·9–48·5
**2**	71	**0**	0–6·4	**100**	91·1–100	**–**	**–**	**41·3**	32·6–50·6
**3**	68	**1·5**	0·1–9·0	**100**	91·1–100	**100**	5·4–100	**42·7**	33·7–52·2
**4**	63	**4·8**	1·2–1·4	**100**	91·1–100	**100**	31·0–100	**45·5**	36·0–55·2
**5**	63	**11·1**	5·0–22·2	**100**	91·1–100	**100**	56·1–100	**47·2**	37·5–57·1
**6**	58	**22·4**	12·9–35·6	**100**	91·1–100	**100**	71·7–100	**52·6**	42·2–62·9
**7**	57	**31·0**	19·9–44·7	**100**	91·1–100	**100**	78·1–100	**55·6**	44·7–65·9
**8**	49	**40·8**	27·3–55·7	**100**	91·1–100	**100**	80·0–100	**63·3**	51·6–73·6
**9**	51	**56·9**	42·3–70·4	**100**	91·1–100	**100**	85·4–100	**69·4**	57·3–79·5
**10**	59	**69·5**	56·0–80·5	**100**	91·1–100	**100**	89·3–100	**73·5**	61·2–83·2
**11**	58	**77·6**	64·4–87·1	**100**	91·1–100	**100**	90·2–100	**79·4**	67·0–88·1
**12**	61	**85·2**	73·3–92·6	**100**	91·1–100	**100**	91·4–100	**84·7**	72·5–92·4
**13**	63	**90·5**	79·8–96·1	**100**	91·1–100	**100**	92·1–100	**89·3**	77·4–95·6
**14**	65	**92·3**	82·2–97·1	**100**	91·1–100	**100**	92·5–100	**90·9**	79·3–96·6
**15**	65	**93·4**	86·2–98·8	**100**	91·1–100	**100**	92·7–100	**94·3**	83·4–98·5
**16**	67	**97·0**	88·7–99·4	**100**	91·1–100	**100**	93·0–100	**96·2**	85·7–99·3
**17**	67	**97·0**	88·7–99·4	**100**	91·1–100	**100**	93·0–100	**96·2**	85·7–99·3
**18**	69	**97·1**	88·8–99·5	**100**	91·1–100	**100**	93·1–100	**96·2**	85·7–99·3
**19**	69	**99·0**	93·7–99·9	**100**	91·1–100	**100**	95·3–100	**98·0**	88·2–99·9
**20**	69	**99·0**	93·7–99·9	**100**	91·1–100	**100**	95·3–100	**98·0**	88·2–99·9
**21**	69	**99·0**	93·7–99·9	**100**	91·1–100	**100**	95·3–100	**98·0**	88·2–99·9
**22**	68	**100**	93·3–100	**100**	91·1–100	**100**	93·3–100	**100**	91·1–100
**23**	68	**100**	93·3–100	**100**	91·1–100	**100**	93·3–100	**100**	91·1–100
**24**	69	**100**	93·4–100	**100**	91·1–100	**100**	93·4–100	**100**	91·1–100
**25**	69	**100**	93·4–100	**100**	91·1–100	**100**	93·4–100	**100**	91·1–100
**26**	69	**100**	93·4–100	**100**	91·1–100	**100**	93·4–100	**100**	91·1–100
**27**	69	**100**	93·4–100	**100**	91·1–100	**100**	93·4–100	**100**	91·1–100
**28**	69	**100**	93·4–100	**100**	91·1–100	**100**	93·4–100	**100**	91·1–100
**29**	69	**100**	93·4–100	**100**	91·1–100	**100**	93·4–100	**100**	91·1–100
**30**	69	**100**	93·4–100	**100**	91·1–100	**100**	93·4–100	**100**	91·1–100
**31**	69	**100**	93·4–100	**100**	91·1–100	**100**	93·4–100	**100**	91·1–100

N, number of COVID positive patients with available serum to be tested on the investigated day.

PPV, Positive predictive value; NPV, Negative predictive value; CI95%, confidence interval at 95%

The cumulative seroconversion curve with respect to days from RT–PCR testing, IgM/IgG positive results were observed in 95.1% at 8 days, as assessed in 62 patients with available sera at 8 days (Table S3). At day 4 post hospitalization, 70.3% of the patients had either IgM and/or IgG positive bands (Figure 4B, and Table S3).

Overall, in this epidemic context, test specificity was 100% irrespective of the delay between symptom onset and serological testing, yielding a 100% positive predicting value (PPV). As expected for a serological test, sensitivity depended on the delay after symptoms appeared. Sensitivity was 56.9% at day 9 after symptom appearance and 97.0% at 16 days post-symptom (Table 2), corresponding to day 9 of hospitalization for nearly all (96.8%) patients (Table S3).

## Discussion

The current SARS-CoV2 pandemic is causing an unprecedented worldwide health crisis that only widespread testing, a goal that has been elusive in many countries, may be able to solve. To that end, validated tools that make COVID-19 testing easier, safe, and faster are welcome additions to the diagnostic landscape. The results of this first bedside fingerprick rapid test in nearly 150 patients demonstrate that the NG test IgG/IgM COVID All-in-One immunoassay can confirm infection in less than 15 min and can be performed by any medical practitioner without needing specialized training or the use of a pathology lab.

Though the test’s sensitivity was low (31.0%) 1-week after symptoms first appeared, this does not necessarily negate its clinical utility for diagnosis. Many patients do not present for days into their illness because their symptoms seem insufficiently severe to access care during a pandemic (per many countries’ national recommendations). In our study population, hospital admission generally occurred 5 days after patients’ initial symptoms appeared. Our immunoassay was able to detect specific antibodies in only 16.8% of patients on day 5 of symptoms, but the fact that it was able to do so in 15 min (as compared to several hours or days for molecular testing) suggests that the test could be a useful tool for triaging patients, especially in overwhelmed hospital settings in high burden areas.

Moreover, seroconversion rates for IgG/IgM increased rapidly during the first two weeks after symptoms appeared, with a cumulative seropositive rate of 50% on the 9^th^ day and 95% at 15 days after a patient became symptomatic. These results are compatible with those recently published using ELISA to detect IgM and IgG [22,23]. The NG All-in-One test also had a sensitivity of >95% at 15 days post-symptom appearance and no false positive results, making it a potentially game changing diagnostic tool in the currently limited arsenal with which to fight the disease.

The NG immunoassay could also serve as a valuable complementary diagnostic to other tests. Despite the high analytical sensitivity of gold standard viral RNA detection, its clinical sensitivity is less than 70% [10,18]. This is perhaps because of poorly performed nasopharyngeal sampling or, when patients access care later at a more serious stage of illness, because false results occur when immune response is high and viral loads lower. For those hospitalized in dedicated COVID-19 wards or in COVID-19 free wards, false results have clinical consequences for exposure and outbreak management. Chest imaging can offset PCR’s lack of sensitivity, but in areas where flu or other respiratory viruses are still circulating, SARS-CoV-2 images can also be misread as viral pneumonia [19,29]. CT and CXR equipment also demand staff and sterilization measures that a simpler bedside rapid test does not [29].

PCR testing’s myriad challenges make testing and diagnosis one of the key bottlenecks to context-adapted, rapid outbreak response. Our study provides robust evidence that: (1) the acute antibody response in SARS-CoV-2 patients are very similar to many other acute viral infections, most importantly SARS-CoV-1 [30] (2) serological testing can be a powerful approach in achieving a timely diagnosis when the test is performed >15 days after symptoms appear [23] and (3) that the time between anti-SARS-CoV-2 IgM and IgG appearance is very short (1–3 days), similar to what was observed for SARS-CoV-1 [30].

Anti-SARS-CoV-2 serology may play a crucial role in the diagnosis of suspected patients at their initial evaluation or for clinically diagnosed patients whose illness has not been confirmed by RNA testing. It may also increase physicians’ confidence when making a COVID-19 diagnosis for two other groups: (i) a healthy, close contact of confirmed COVID-19 cases during the quarantine period that would be deemed a probable carrier if antibody positive (especially because RNA testing is not performed for mild or asymptomatic patients) and (ii) RNA confirmed seropositive patients that have specific antibodies have been induced and likely produced immunity.

It has been less than three months since SARS-CoV-2 first invaded humans, and the prevalence of anti-SARS-CoV-2 antibodies is nearly zero. Therefore, in the current outbreak (that will likely to continue for months), seropositive individuals could be a probable preceding infector. Presence of IgM could be considered as a recent infection marker similar, while IgG follow up as a likely indicator of immunity [30]. If, SARS-CoV-2 becomes an enduring respiratory pathogen in humans like influenzas or other less-pathogenic coronaviruses (rather than able to be eradicated like SARS-CoV-1), serological diagnosis of acute SARS-CoV-2 infection will depend on IgM detection in post-epidemic areas in subsequent epidemic seasons.

Unlike other studies using ELISA for serology, we did not see a correlation between a seroconversion delay and clinical severity. This is likely because our test provides a positive/negative result and does not allow for IgM/IgG titration. In a recent study, authors suggested that higher antibody titres may be a risk factor for critical illness, independent of older age, male gender, and comorbidities [23]. In our study, the NG test IgG/IgM COVID All-in-one was read at 15 min, but it is obvious that in most of the IgM + IgG positive cases the signals appeared within ≤2 min. This may allow the evaluation of antibody-dependent disease enhancement effects, like those commonly found in SARS-CoV-1 patients [30,32].

Our study presented some limitations: (1) RT–PCR detection was based on upper respiratory tract specimens from patients with severe symptoms. None were asymptomatic (those patients did not access care). (2) Most study patients’ diagnoses were based on positive RT–PCR results that used respiratory samples. Patients with negative RT–PCR but with chest imaging compatible with COVID-19 were not included. (3) Because the epidemic in France is very recent (1 month), samples were collected during the acute phase of illness. Accordingly, we don’t yet have sera from later stages to evaluate the persistence of antibodies then. (4) Even though specificity is excellent in the studied patients (including 4 COVID negative patients with other coronaviral infections), these tests should be evaluated with more non-COVID-19 coronaviral infections to definitively establish the cross-reactivity of the assay.

## Conclusion

This assessment demonstrates that serological testing has critical value as an initial diagnostic assay and a complement to direct RNA testing. It provides evidence for the routine application of serological testing in the diagnosis and clinical management of COVID-19 patients. The NG test achieved a sensitivity of >95% after 15 days and a 100% specificity (no false positives; PPV of 100%) for the period after symptoms appear. The NG-Test IgM-IgG COVID All-in-One assay is simple, cheap, rapid, easy to interpret, and practical (can be stored at room temperature). It reliably detects IgM & IgG and can be performed directly at a patient’s bedside at a general physician’s office, or when triaging in an emergency department. No observable difference was seen when using a single drop of whole blood (at the bedside of the patient) versus 10 µl of serum in a pathology laboratory (T. Naas, personal comm).

The main limitation of serological testing is the fact that, after symptoms appear, sensitivity directly depends on the day that the test is conducted, with low sensitivity for the first days of infection when RT–PCR is more accurate. However, our test might be more useful over the longer term. Though antibodies are likely involved in the clearance of the primary infection [21], individuals who survive SARS-CoV-2 are likely to possess neutralizing antibodies protecting them from possible re-infection, as observed with SARS-CoV-1 where >90% of patients had detectable IgGs 2-years after infection [31–33]. Thus, our immunoassay could be used to follow healthcare workers in daily contact with infected patients. Determining their immunity status may not reduce mandatory precautions for working with COVID-19 patients, but it may reduce the fear of infection when in close contact with the virus. Furthermore, this test may allow non-medical essential workers (such as law enforcement officers, supermarket and post office employees, funeral home, burial, and nursing home staff) who continue to work during community social isolation periods to be monitored serologically. These tests will also be critical for the period after social distancing measures end and the serological status of the general population will need to be understood in order to identify those with immunity and those requiring further protective means. In addition, these sorts of tests have shown their usefulness to evaluate the population level antibody prevalence, including one US county (Santa Clara: 2.49%−4.16%) where infections were 85-fold more widespread than indicated by confirmed cases [34]. These data are crucial to calibrate epidemic and mortality projections. Finally, this test may also be useful for the many patients who are hospitalized more than 8 days after milder symptoms first appear and could serve as confirmation of infection for those who with negative PCR results and imaging typical of viral pneumonia. The test could be performed directly by physicians to confirm COVID-19.

## Supplementary Material

revised-DORTET-et-al-EMI-COVID-19-Suppl-Figures_and_tables.docx
