# Supplementary material for: Rapid Determination of SARS-CoV-2 antibodies using a bedside, point-of-Care, serological test
Source: Emerg Microbes Infect. 2020 Oct 7;9(1):2212–21. doi: 10.1080/22221751.2020.1826892 (PMC7580567; doi:10.1080/22221751.2020.1826892)
Supplement: revised-DORTET-et-al-EMI-COVID-19-Suppl-Figures_and_tables.docx [file TEMI_A_1826892_SM5361.docx]

Revised TEMI-2020-0792 R2

**Supplementary figures and tables**

Rapid Determination of SARS-CoV-2 Antibodies Using a Bedside, Point-of-Care, Serological Test

Laurent DORTET^1^_,_ Cecile EMERAUD,^1^ Christelle VAULOUP-FELLOUS,^2^ Mouna KHECHAREM,^1^ Jean-Baptiste RONAT,^3^ Nicolas FORTINEAU,^1^ Anne-Marie ROQUE-AFONSO,^2^ and Thierry NAAS^1^

^1^ Service *de Bactériologie-hygiène, Hôpital Bicêtre, Inserm U 1184 ; LabEx LERMIT, Université Paris-Saclay Le Kremlin-Bicêtre ; ^2^ Service de Virologie, Hôpital Paul-Brousse,* *Inserm U 1193 ; Université Paris-Saclay Villejuif, APHP Paris-Saclay, France*

^3^ Médecins Sans Frontières, Mini-Lab project, Paris, France

**SUPPLEMENTARY FIGURES**

**Supplementary Figure S1.** Technical manual of the NG-Test IgM-IgG COVID All-in-One

**SUPPLEMENTARY TABLES**

**Table S1:** Number of sera per patient

| **Number of sera per patient** | **Number of patients** |
| --- | --- |
| 1 serum | 21 |
| 2 sera | 15 |
| 3 sera | 56 |
| 4 sera | 8 |
| 5 sera | 1 |

**Table S2.** Kinetic for individual results of the NG-Test IgM-IgG COVID All-in-One for 101 SARS-CoV-2 RT-PCR positive patients

**
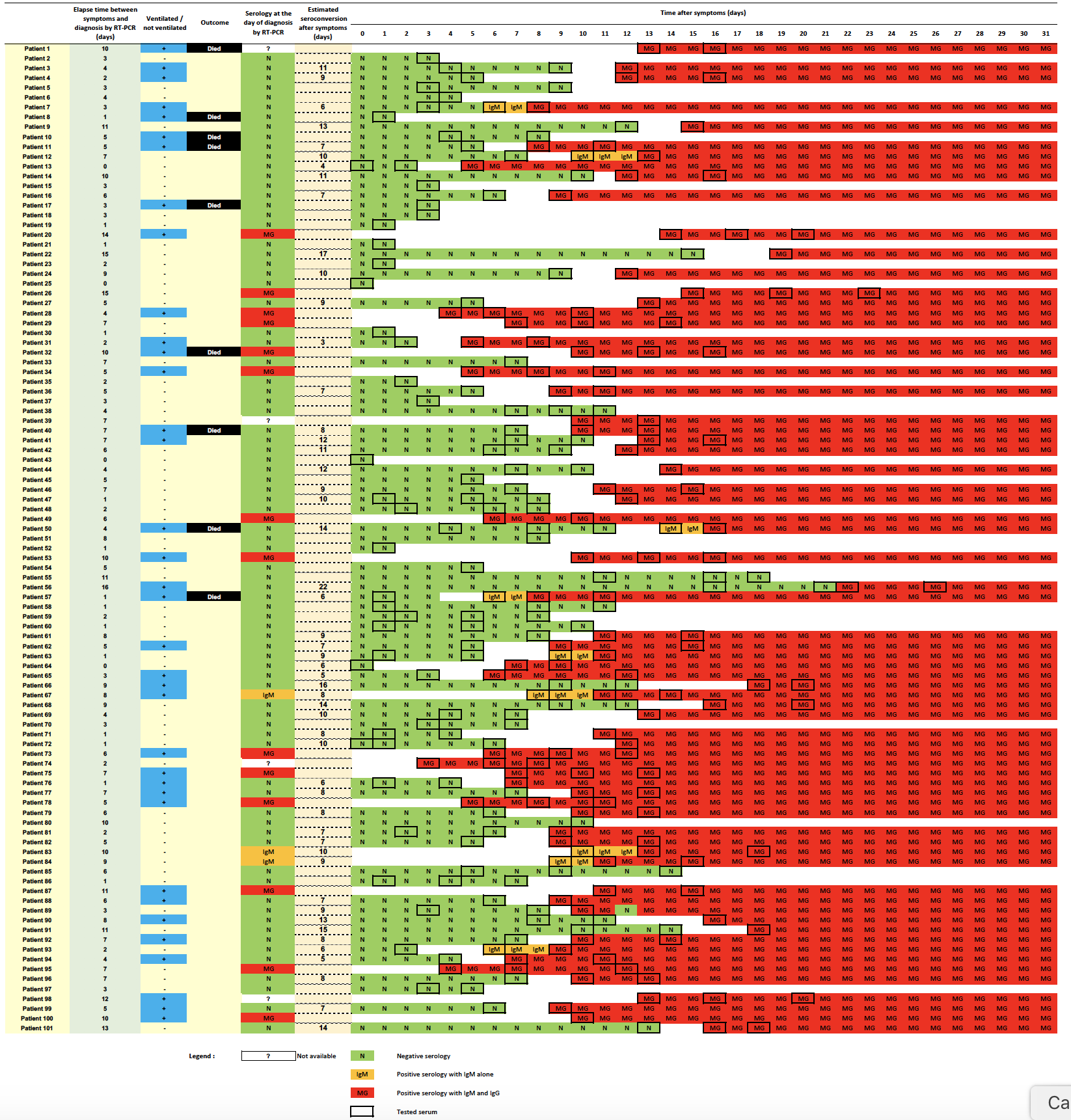
**

**Table S3.** Performance of the NG-Test IgM-IgG COVID All-in-One by day of diagnosis by RT-PCR

| **Day after diagnosis by RT-PCR** | **N^a^** | **Cumulative # of patients** | | **Sensitivity** | | | **Specificity** | | **PPV** | | **NPV** | |
| --- | --- | --- | --- | --- | --- | --- | --- | --- | --- | --- | --- | --- |
|  |  | **Sero +^b^** | **Total ^c^** | **%** | **CI95 %** | **%** | | **CI95 %** | **%** | **CI95 %** | **%** | **CI95 %** |
| **-3** | 82 | 1 | 82 | **1.2** | 0.06 - 7.5 | **100** | | 91.1 - 100 | **100** | 5.5 - 100 | **38.2** | 29.9 - 47.1 |
| **-2** | 82 | 1 | 82 | **1.2** | 0.06 - 7.5 | **100** | | 91.1 - 100 | **100** | 5.5 - 100 | **38.2** | 29.9 - 47.1 |
| **-1** | 82 | 1 | 82 | **1.2** | 0.06 - 7.5 | **100** | | 91.1 - 100 | **100** | 5.5 - 100 | **38.2** | 29.9 - 47.1 |
| **0** | 96 | 17 | 97 | **17.5** | 10.1 - 26.0 | **100** | | 91.1 - 100 | **100** | 75.9 - 100 | **38.5** | 30.2 - 47.4 |
| **1** | 52 | 18 | 99 | **34.6** | 22.3 - 49.2 | **100** | | 91.1 - 100 | **100** | 78.1 - 100 | **59.5** | 48.2 - 69.9 |
| **2** | 51 | 19 | 100 | **37.3** | 24.5 - 51.9 | **100** | | 91.1 - 100 | **100** | 79.1 -100 | **61.0** | 49.5 - 71.4 |
| **3** | 65 | 36 | 101 | **55.4** | 42.6 - 67.5 | **100** | | 91.1 - 100 | **100** | 88.0 - 100 | **63.3** | 51.6 - 73.6 |
| **4** | 64 | 45 | 101 | **70.3** | 57.4 - 80.8 | **100** | | 91.1 - 100 | **100** | 90.2 - 100 | **72.5** | 60.2 - 82.2 |
| **5** | 62 | 47 | 101 | **75.8** | 63.0 - 85.4 | **100** | | 91.1 - 100 | **100** | 90.6 - 100 | **76.9** | 64.5 - 86.1 |
| **6** | 62 | 50 | 101 | **80.6** | 68.2 - 89.2 | **100** | | 91.1 - 100 | **100** | 91.1 - 100 | **80.6** | 68.3 - 89.2 |
| **7** | 62 | 55 | 101 | **88.7** | 77.5 - 95.0 | **100** | | 91.1 - 100 | **100** | 91.9 - 100 | **87.7** | 75.7 - 94.5 |
| **8** | 62 | 59 | 101 | **95.2** | 85.6 - 98.7 | **100** | | 91.1 - 100 | **100** | 92.4 - 100 | **94.3** | 83.3 - 98.5 |
| **9** | 63 | 61 | 101 | **96.8** | 88.0 - 99.4 | **100** | | 91.1 - 100 | **100** | 92.6 -100 | **96.2** | 85.7 - 99.3 |
| **10** | 68 | 67 | 101 | **98.5** | 90.9 - 99.9 | **100** | | 91.1 - 100 | **100** | 93.1 -100 | **98.0** | 88.2 - 99.9 |
| **11** | 68 | 68 | 101 | **100** | 93.2 - 100 | **100** | | 91.1 - 100 | **100** | 93.2 - 100 | **100** | 91.1 - 100 |

**^a^***N, number of COVID positive patients with available serum results on the investigated day;* *^b^cumulative number of patients with a positive serology; ^c^cumulative total number of patients tested*

*PPV, Positive predictive value; NPV, Negative predictive value; CI95%, confidence interval at 95%*
